# Partitioning of copy-number genotypes in pedigrees

**DOI:** 10.1186/1471-2105-11-226

**Published:** 2010-05-03

**Authors:** Louis-Philippe Lemieux Perreault, Gregor U Andelfinger, Géraldine Asselin, Marie-Pierre Dubé

**Affiliations:** 1 Montreal Heart Institute Research Center, 500, Bélanger Street, Montréal, Canada; 2 Université de Montréal, 2900, chemin de la tour, Montréal, Canada; 3 CHU Sainte-Justine, 3175, Chemin de la Côte-Sainte-Catherine, Montréal, Canada

## Abstract

**Background:**

Copy number variations (CNVs) and polymorphisms (CNPs) have only recently gained the genetic community's attention. Conservative estimates have shown that CNVs and CNPs might affect more than 10% of the genome and that they may be at least as important as single nucleotide polymorphisms in assessing human variability. Widely used tools for CNP analysis have been implemented in *Birdsuite *and *PLINK *for the purpose of conducting genetic association studies based on the unpartitioned total number of CNP copies provided by the intensities from Affymetrix's Genome-Wide Human SNP Array. Here, we are interested in partitioning copy number variations and polymorphisms in extended pedigrees for the purpose of linkage analysis on familial data.

**Results:**

We have developed *CNGen*, a new software for the partitioning of copy number polymorphism using the integrated genotypes from *Birdsuite *with the Affymetrix platform. The algorithm applied to familial trios or extended pedigrees can produce partitioned copy number genotypes with distinct parental alleles. We have validated the algorithm using simulations on a complex pedigree structure using frequencies calculated from a real dataset of 300 genotyped samples from 42 pedigrees segregating a congenital heart defect phenotype.

**Conclusions:**

*CNGen *is the first published software for the partitioning of copy number genotypes in pedigrees, making possible the use CNPs and CNVs for linkage analysis. It was implemented with the *Python *interpreter version 2.5.2. It was successfully tested on current Linux, Windows and Mac OS workstations.

## Background

Copy number variations (CNVs) and polymorphisms (CNPs) have recently gained much interest as a novel tool to study the relationship between genomic variation and disease. CNVs and CNPs are widespread throughout the genome and were shown to be largely heritable while being responsible for a significant amount of inter-variability in human [1]. They can also appear *de novo *at a significant rate, both in germline and somatic cells [2]. Any variation in copy number has the possibility of affecting a wide spectrum of genes, which might lead to genomic disorders [3]. Variation in gene-expression levels can occur for genes located within a region of copy number variation [2], and negative correlations between CNV and gene expression were reported in approximately 10% of cases [4]. It is currently estimated that up to 12% of the genome is subject to copy number variations [5,6]. Those genetic variations are likely to play an important role in the etiology of common disease and sporadic birth defects [1], partly attributable to their higher mutation rate as compared to point mutation [7] and due to their considerable genomic coverage.

High-density SNP genotyping arrays are commonly used for CNV/CNP analysis. Those arrays provide signal intensities of alleles across all SNPs which can be used to infer copy numbers along with a selection of CNV-specific probes. The presence of a CNV/CNP region has the potential to confuse SNP calling algorithms if unaccounted for, as SNPs can be represent with multiple or single alleles. It is then crucial to gain knowledge of CNV and CNP in genetic analysis, even when using SNPs as a marker.

While amenable to genetic association studies, the use of CNVs and CNPs in linkage analysis with multi-generational family data has up to now been greatly limited by the requirement of chromosome-specific copy number assignments, which, to our knowledge, none of the current software indexed in the literature is able to provide. Multi-allelic partitioned copy number polymorphisms have the potential to offer a new and powerful tool for linkage analysis. Today's high density SNP panels offer near-optimal coverage for linkage analysis. However, some regions, especially those with copy number polymorphisms, may have been less well covered due to the requirements of Mendelian consistency prior to linkage analysis. Although representing only a minute fraction of the genome, the partitioning of copy number genotypes has the potential to help fill-in the remaining linkage coverage gaps.

The use of genome-wide association studies (GWAS) with unrelated cases and controls is a popular approach for the discovery of genetic variants responsible for common genetic diseases [8]. Linkage analysis with extended pedigrees is of limited use for the identification of common polymorphisms of low effect, but it does offer high detection power with more penetrant variants even in the presence of multiple rare causal variants at a single locus [9] or highly penetrant rare variants throughout the genome. Furthermore, the combined use of pedigree-based linkage analysis and association studies in a multistage approach was argued by Elston *et al. *to be both powerful and advantageous [9]. Significantly linked markers can emphasize candidate genes for subsequent association study and information on candidate loci can be incorporated into association tests using either a generalized logistic regression [10] or a quantitative linkage score [11].

Here, we are interested in using CNV and CNP data from the Affymetrix 6.0 chip analyzed with the *Fawkes *program of the *Birdsuite *software [12,13]. *Fawkes *creates an integrated genotype from SNPs, rare copy number variations and common copy number polymorphisms genotypes information, providing the number and type (*A *or *B*) of each allele for each SNP on the Affymetrix Genome-Wide Human SNP Array 5.0 and 6.0 chips. While the suite comes with *Python *scripts for file compatibility with the whole-genome association toolset PLINK [14], no software is available to conduct chromosome assignment of the copy number genotypes based on pedigree information. We propose a new algorithm called *CNGen *that uses SNP genotypes in multi-generational pedigrees to convert *Fawkes*' genotypes into partitioned copy number genotypes (CN genotypes) which can then be treated as multi-allelic markers by common linkage software such as *MERLIN *[15]. We have developed *Python *scripts to encode CN genotypes into multi-allelic genotypes. We have validated and successfully applied the algorithm in the analysis of multi-generational pedigrees through simulation procedures.

## Implementation

The standard *Fawkes *output file is tabulated with samples in columns and probe sets (SNPs) in rows. Each cell contains a *Fawkes *call that is a comma-separated value of the form [*a, b*] where *a *is the number of copies of allele *A *and *b*, the number of copies of allele *B*. Five different *Fawkes *calls are possible:

1. undefined calls, from a probe set of the form [-1, -1] that was unresolved by *Fawkes*;

2. heterozygous calls of the form [*a*, *b*] where *a *and *b *≠ 0;

3. null calls of the form [0, 0], representing a null genotype;

4. hemizygous calls of the form [0, *b*] or [*a*, 0], where *a *and *b *= 1;

5. homozygous calls, of the form [0, *b*] or [*a*, 0], where *a *and *b *> 1.

*CNGen *converts *Fawkes *calls into partitioned CN genotypes as comma-separated values of the form [*T*_1_*m, T*_2_*n*] where *T*_*i *_is the allele type (one of *A*, *B *or *N *for null) on one of the parental chromosome, and *m *and *n *represent the number of copies of the named allele type on the specified chromosome. The *N *allele type represents an absence of either an *A *or *B *allele on a given parental chromosome. The partitioning of copy numbers is accomplished according to the rules of Mendelian transmission and under the general assumption that ancestral copy number expansions were of the same allele type, *i.e. *a copy number expansion from 1 to 2 copies is not allowed to bear both *A *and *B *alleles on the same chromosome strand.

This last assumption affects only copy numbers of two or more, since single-copy alleles will result in one copy which will by default be located on a single chromosome. Situations with two copies where the true CN genotype is [*A*2, *N*], [*B*2, *N*] and [*A*1, *B*1] will be appropriately called. However true [*A*1*B*1, *N*] will not and will likely give rise to Mendelian inconsistencies which will be coded as undefined by the *CNGen *algorithm. Expansions beyond 2 copies were found less frequently than 0, 1, and 2 copies by a survey of 300 genotyped individuals in 42 pedigrees presenting a congenital heart defect phenotype. Overall, only 0.07% of *Fawkes *calls had three or more copies (expansions), compared to 2.2% with 0 or 1 copy (deletions) and 97.6% with 2 copies, with the rest being undefined *Fawkes *calls.

### Step 1 - Partitioning of non-homozygous calls

The algorithm begins by parsing the *Fawkes *calls to generate in this first pass the CN genotypes for the first four of the five possible *Fawkes *calls. Undefined and null *Fawkes *genotypes are set to undefined or null CN genotypes, respectively. For single hemizygous *Fawkes *genotype, the first chromosome is set to hold the deletion (*N*) and the other, the given allele (*A *or *B*). Finally, heterozygous *Fawkes *calls are partitioned such that each chromosome receives the copies of only one allele type. Those conversions from *Fawkes *genotypes to partitioned CN genotypes are summarized in Table 1.

**Table 1:** Direct Fawkes conversion. Example of direct conversion from integrated genotypes (*Fawkes *to CN genotypes. The type-1 homozygous genotypes are converted using information from first-degree relatives with one of those *Fawkes *calls: heterozygous, null or hemizygous.

*Fawkes *genotypes		CN genotypes
	Undefined	
-1, -1	→	-1, -1

	Heterozygous	
*a, b *(*a *and *b *≠ 0)	→	*Aa, Bb*

	Null	
0, 0	→	*N, N*

	Single hemizygous	
1, 0	→	*A*1, *N*
0, 1	→	*B*1, *N*

### Step 2 - Partitioning of type-I homozygous calls

**Figure 1:**
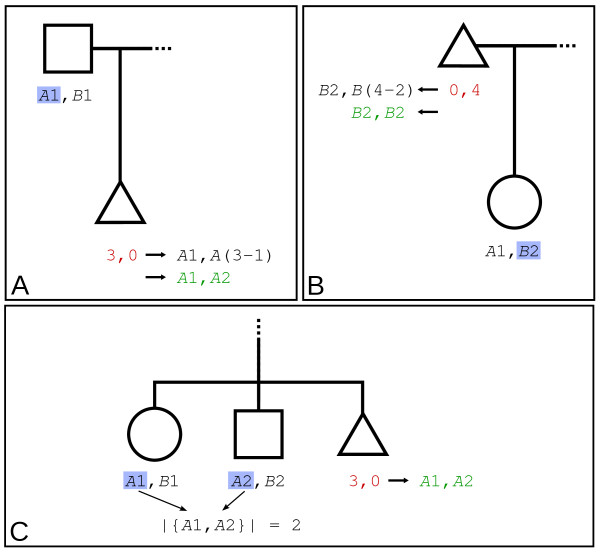
**Type-I homozygous conversion**. Representation of the conversion of Type-I homozygous *Fawkes *genotypes (red) into partitioned CN genotypes (green). The triangle represents the index individual (*I*). (A) Conversion of *I*'s *Fawkes *genotype using one heterozygous parent, (B) one heterozygous child or (C) two heterozygous siblings. In each case, the sex of the individual used for conversion is not important.

We distinguish two types of homozygous *Fawkes *calls based on the genotype conversion method used: type-I and type-II. CN genotype partitioning for type-I homozygous *Fawkes *calls is solved by relying on information from a heterozygous first-degree relative and assuming Mendelian transmission. The algorithm searches for heterozygous first-degree relatives (parents, children and siblings) of the index individual to be converted (*I*), as those will have partitioned CN genotypes that can be used as reference. Figure 1 presents the different scenarios for type-I homozygote partitioning.

#### Step 2a

When a parent of *I *has a heterozygous CN genotype of the form [*Am*_*p*_, *Bn*_*p*_], then *I *is assigned the following CN genotype:(1)

where *I*_*F*, *L *_and *I*_*CN*, *L *_are the *Fawkes *genotype and the new CN genotype of the index individual at locus *L*, respectively (Figure 1A). If *a *- *m*_*p *_or *b *- *n*_*p *_equals 0, the second partitioned CN allele becomes *N*.

#### Step 2b

If *I *does not have a heterozygous parent, the algorithm searches for the presence of a child with a heterozygous CN genotype of the form [*Am*_*c*_, *Bn*_*c*_]. The partition of the CN genotype is solved as presented in Equation (1) by replacing *m*_*p *_and *n*_*p *_by *m*_*c *_and *n*_*c *_respectively (Figure 1B).

#### Step 2c

If *I *does not have a heterozygous child, then the algorithm searches for the presence of two siblings with distinct heterozygous CN genotypes [*Am*_*s*1_, *Bn*_*s*1_] and [*Am*_*s*2_, *Bn*_*s*2_] for which the cardinality of the pool of CN alleles of the same type as *I *is two, *i.e. *(|{*Am*_*s*1_, *Am*_*s*2_}| = 2 if *I*_*F*, *L *_= [*a*, 0], *m*_*s*1 _≠ *m*_*s*2_) or (|{*Bn*_*s*1_, *Bn*_*s*2_}| = 2 if *I*_*F*, *L *_= [0, *b*], *n*_*s*1 _≠ *n*_*s*2_). Then, *I *is assigned the following CN genotype:(2)

Restricting the conditions *m*_*s*1 _≠ *m*_*s*2 _or *n*_*s*1 _≠ *n*_*s*2_, ensures that both CN alleles originate from the two distinct parents (Figure 1C). Any *Fawkes *homozygous calls that remain un-converted are then flagged as type-2 *Fawkes *homozygous calls and the algorithm proceeds to step 3.

### Step 3 - Partitioning of type-II homozygous calls

**Figure 2:**
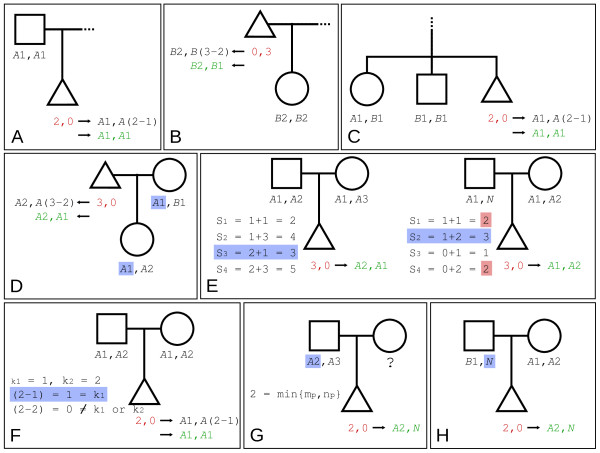
**Type-II homozygous conversion**. Representation of the conversion of Type-II homozygous *Fawkes *genotypes (red) into partitioned CN genotypes (green). The triangle represents the index individual (*I*). (A) Conversion of *I*'s *Fawkes *genotype using one homozygous parent or (B) using one homozygous child. (C) represents the conversion using two sibs: one heterozygote and one homozygote. (D) uses a child and the spouse of the index individual. (E) uses the two homozygous parents of *I*. The pedigree on the left shows the conversion when there are 4 different sums, and the pedigree on the right, when there are only 3 different sums (see step 3e above). Panel (F) shows the conversion when two homozygous parents with the same genotype is used. Finally, the conversion methods when one parent has a unknown genotype or a null allele are shown in panel (G) and (H) respectively.

CN genotype partitioning of type-II homozygous *Fawkes *calls proceeds by assuming Mendelian transmission of CN alleles and by relying on information in the nuclear pedigree of *I*. The algorithm searches for a solution according to the following sequential attempts. Figure 2 presents the different scenarios for type-II homozygous partitioning.

#### Step 3a

First, the algorithm searches for the presence of one parent of *I *that is homozygous for a CN genotype of the same allele-type as *I *such as [*T*_1_*m*_*p*_, *T*_2_*n*_*p*_] where *T*_1 _= *T*_2_, *T*_*i *= 1,2 _∈ {*A*, *B*, *N*} and *m*_*p *_= *n*_*p*_; in which case *I *is assigned its CN genotype according to Equation (1) (Figure 2A).

#### Step 3b

Analogously to step 3a above, a child of *I *presenting a homozygous CN genotype [*T*_1_*m*_*c*_, *T*_2_*n*_*c*_] can be used according to Equation (1) (*m*_*c *_replacing *m*_*p *_and *n*_*c*_, *n*_*p*_) (Figure 2B).

#### Step 3c

If no such parent or child exists, the algorithm then searches for the presence of two siblings, one with a heterozygous genotype of the form [*Am*_*s*1_, *Bn*_*s*1_], and the other one with a homozygous CN genotype with identical CN alleles excluding null alleles and of a different allele-type than that of *I *(*i.e. *[*Am*_*s*2_, *An*_*s*2_] if *I*'s *Fawkes *genotypes is [0, *b*] or [*Bm*_*s*2_, *Bn*_*s*2_] if *I*'s *Fawkes *genotypes is [*a*, 0]). In this case, *I*_*CN*, *L *_is defined by Equation (1) with replacement of *m*_*p *_and *n*_*p *_by *m*_*s*1 _and *n*_*s*1_, respectively (Figure 2C).

#### Step 3d

If *I*'s genotype at the given locus remains unconverted, the algorithm searches for the presence of one child and the spouse of *I *where both have distinct CN genotypes. In this case, one CN allele is obligatorily shared between the child and the spouse and the remaining CN allele of the child can be assigned to *I*. The spouse's CN genotype has the form [*T*_1_*m*_*s*_, *T*_2_*n*_*s*_] and the child's CN genotype [*T*_1_*m*_*c*_, *T*_2_*n*_*c*_] where |{*T*_1_*m*_*s*_, *T*_2_*n*_*s*_} ∩ {*T*_1_*m*_*c*_, *T*_2_*n*_*c*_}| = 1. *I *is assigned the remaining child's CN allele according to {*T*_1_*m*_*c*_, *T*_2_*n*_*c*_} - {*T*_1_*m*_*s*_, *T*_2_*n*_*s*_} and the algorithm infers the other CN allele of *I *(Figure 2D).

#### Step 3e

If the algorithm has not yet converted the *Fawkes *genotype according to the above steps, it then searches for cases where the two parents of *I *are both homozygotes of the same allele type as *I *but with distinct CN genotypes. Here, one parent's genotype can be inferred if its CN genotype is undefined. A solution exists if the first parent has a CN genotype of the form [*T*_1_*m*_*p*1_, *T*_2_*n*_*p*1_] and the second parent, [*T*_1_*m*_*p*2_, *T*_2_*n*_*p*2_], where *m*_*p*1 _≠ *m*_*p*2 _or *n*_*p*1 _≠ *n*_*p*2_. In both cases, *T*_1 _= *T*_2 _= *A *if *I*_*F*, *L *_= [*a*, 0] and *T*_1 _= *T*_2 _= *B *if *I*_*F*, *L *_= [0, *b*]. Parents may have a null allele on one chromosome. The following sums are then calculated:

If the number of unique sums is 4 (*i.e. *|{*s*_1_, *s*_2_, *s*_3_, *s*_4_}| = 4), the sum *s*_*i *_that corresponds to the *Fawkes *genotype of *I *is used to assign the corresponding parental CN alleles to *I*. If the number of unique sums is 3 (*i.e. *|{*s*_1_, *s*_2_, *s*_3_, *s*_4_}| = 3), then the algorithm checks whether *I*'s *Fawkes *genotype matches the min or max{*s*_1_, *s*_2_, *s*_3_, *s*_4_}, in which case the corresponding parental CN alleles can be assigned to *I *(Figure 2E).

#### Step 3f

If the two homozygous parents have identical CN genotypes of the same allele-type as that of the index individual (first parent having a CN genotype [*T*_1_*m*_*p*1_, *T*_2_*n*_*p*1_] and second parent, [*T*_1_*m*_*p*2_, *T*_2_*n*_*p*2_] where *m*_*p*1 _= *m*_*p*2 _= *k*_1 _and *n*_*p*1 _= *n*_*p*2 _= *k*_2_, *T*_1 _= *T*_2 _= *A *if *I*_*F*, *L *_= [*a*, 0] or *T*_1 _= *T*_2 _= *B *if *I*_*F*, *L *_= [0, *b*] for both parent), then *I *is assigned the CN genotype described in Equation (3) (Figure 2F).(3)

#### Step 3g

If only one parent of *I *has a CN genotype with the same CN allele type as *I *([*T*_1_*m*_*p*_, *T*_2_*n*_*p*_] where *T*_1 _= *T*_2 _= *A *if *I*_*F*, *L *_= [*a*, 0] or *T*_1 _= *T*_2 _= *B *if *I*_*F*, *L *_= [0, *b*]) with the possibility of one null allele, if (*a *+ *b*) = min{*m*_*p*_, *n*_*p*_} = *z*, then *I*_*CN*, *L *_= [*T*_1_*z, N*] (Figure 2G).

#### Step 3h

Finally, if one parent of *I *has a heterozygous CN genotype containing a *N *CN allele and the remaining allele is of a different allele-type as that of *I *(*i.e. *[*Bn*_*p*_, *N *] if *I*_*F*, *L *_= [*a*, 0] or [*Am*_*p*_, *N *] if *I*_*F*, *L *_= [0, *b*]) and if both parents of *I *have a CN genotype for this loci and the trio respects Mendelian transmission (or only one parent is converted or genotyped), the *N *allele is assigned to *I *and the second allele is inferred (Figure 2H).

### General procedure of the algorithm

The developed algorithm reads a *pedfile *in linkage format containing the pedigree structures, and then opens the *Fawkes *output file generated by *Birdsuite*. Reading-in one marker of the *Fawkes *file at a time, *CNGen *begins by converting *Fawkes *genotypes of type 1 to 4 into CN genotypes as described in step 1 (see Table 1). Next, homozygous type-I calls are converted based on heterozygous first-degree relatives (step 2 and Figure 1). Any encountered Mendelian inconsistencies are reported. Unconverted type-I homozygous calls are flagged as type-II. Then, the algorithm attempts to partition the remaining type-II homozygous calls by inspection of the converted first-degree relatives of the index individual according to procedures described in step 3 (Figure 2). Following Mendelian laws, and based on first degree relatives' CN genotypes, obligate genotype assignments are resolved. The algorithm cycles to resolve all unconverted type-II homozygote each time it has successfully partitioned at least one call. When no more calls can be partitioned, remaining *Fawkes *calls and obligate Mendelian inconsistencies are set to a CN genotype of [-2, -2] and the algorithm proceeds to the following marker.

The algorithm outputs a tabulated file containing partitioned CN genotypes following the *Fawkes*' format. A log file is created and summary statistics of the partitioning procedures are sent to the standard output, including the percentage of each type of calls, the percentage of successful conversions and the number of Mendelian inconsistencies found during the process. *CNGen *does not specifically search for all Mendelian errors in the pedigrees but it reports those found during type-I and -II homozygous call conversions (step 2 and 3, respectively). The popular program *PedCheck *[16] can be used to systematically search for Mendelian errors, as per common linkage practice. A companion tool to interface with *PedCheck *was developed.

## Results and Discussion

### Implementation

The *CNGen *algorithm was implemented with the *Python *interpreter version 2.5.2. It was successfully tested on current Linux, Windows and Mac OS workstations. System resource requirements are dependent on the size of the input datasets, proportionally with the number of samples in the analysis. On a modern Linux workstation, the conversion of approximately 273 million calls (909,622 markers from the Affymetrix 6.0 chip for 42 pedigrees [300 individuals]) required less than 10 Mb of RAM and a little more than one hour of computation time. *CNGen *is the first software to produce partitioned copy number genotype from *Birdsuite*'s integrated SNP genotypes. Partitioned CN genotypes offer the valuable possibility of using copy number variation in the context of linkage studies.

### Validation

**Figure 3:**
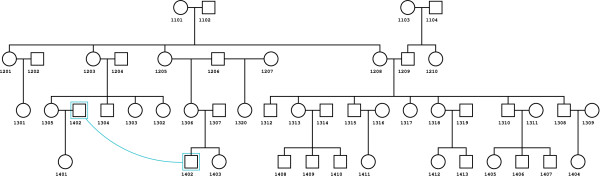
**Complex pedigree for the simulation**. Representation of the complex pedigree used for validation simulation runs. The pedigree has 47 individuals including 14 founders. Individual *1402 *creates a consanguinity loop in the pedigree. The diagram is modified from *Cranefoot*'s resulting pedigree [18].

We have validated the algorithm using simulations on a multi-generational pedigree consisting of 47 individuals including 14 founders (Figure 3). Gene-dropping simulations were generated. First, founders were assigned a null, hemizygote, heterozygote or homozygote CN genotype state following proportions given by real data (~0.0337%, ~2.14%, ~26.9% and ~70.9%, respectively). An allele is then randomly chosen from a set of all possible CN genotypes depending of the given state. Mendelian segregation laws were used to assign CN genotypes to non-founding pedigree members, receiving one random allele from each parent. 1% of all CN genotypes were randomly selected and recoded as undefined CN genotypes ([-1, -1]). CN allele frequencies are presented in Table 2. CN genotypes were then converted into *Fawkes *genotypes based on the number of *A *and *B *alleles (*i.e. *[*Am, Bn*] → [*m, n*], [*Am, An*] → [(*m *+ *n*), 0], [*Bm, N*] → [0, *m*], etc.). Finally, *CNGen *was used to partition the *Fawkes *genotype back into CN genotypes and comparison between the true CN genotypes and the ones inferred by *CNGen *were compared. Three million validation runs were thus completed, for which more than 140 million genotype conversions were made, and which covered every possible conversion step from *Fawkes *to CN genotypes (additional file 1). The validation procedure confirmed that all converted genotypes by *CNGen *were accurate. Irresolvable homozygous type-II calls due to lack of information from first-degree relatives were checked and validated.

**Table 2:** Allele frequencies after simulation. CN allele frequencies after three million simulations, frequencies for 14 founders and all 47 pedigree members are presented.

Alleles	Only Founders		All individuals	
	n	%	n	%
*A*1	12294813	0.146	41275221	0.146
*A*2	10728256	0.128	35999291	0.128
*A*3	6661654	0.0793	22367164	0.0793
*A*4	4068729	0.0484	13667675	0.0485
*A*5	1472417	0.0175	4938921	0.0175
*B*1	12291382	0.146	41262843	0.146
*B*2	10732261	0.128	36029869	0.128
*B*3	6662835	0.0793	22364446	0.0793
*B*4	4068582	0.0484	13652583	0.0484
*B*5	1473733	0.0175	4949877	0.0176
*N*	12706756	0.151	42672446	0.151
-1	838582	0.00998	2819664	0.01

**Total**	**84000000**		**282000000**	

For an additional 30,000 validation runs (additional file 2), we substituted the CN genotype of a random pedigree member with a different randomly selected CN genotype and allowed 10% of CN genotypes to be recoded as undefined CN genotypes ([-1, -1]). Following *CNGen*, we ran *PedCheck *and nuclear families where inconsistent transmissions were found were set to missing. Overall, 81% of the inserted CN genotype errors were detected by the process. 59% of 30,000 simulations resulted in the concerned nuclear family being detected by *PedCheck*. In 22% of the 30,000 simulation runs, *CNGen *had assigned an undefined CN genotype at the modified individual. *CNGen *and *PedCheck *assigned an undefined value to respectively 14% and 7% of the 1,410,000 calls for a total of 21% of undefined calls. Overall, only 5,506 out of the 30,000 simulations (18.4%) resulted in a wrong CN genotype assignment to the substituted individual or to his first-degree relatives, representing an undetected genotype error rate of ~0.5% for 1,410,000 calls (30,000 simulations × 47 individuals) with a simulated 2.13% genotyping error rate. In a typical study exposed to a 1% genotyping error rate, this would result in 0.2% of undetected genotype errors. These findings confirm that CNGen will not result in an excess of false calls in the presence of erroneous or *de novo *CNP.

## Conclusions

*CNGen *is, to our knowledge, the first software that allows the partitioning of copy number genotypes in extended pedigrees for the purpose of linkage analysis with CNPs. *CNGen *is a flexible, open source *Python *program that can process integrated SNP genotypes from the *Fawkes *routine of the *Birdsuite *program for high-density SNP genotyping arrays. *Birdsuite *was developed for the Affymetrix's SNP array 5.0 and 6.0, but, as mentioned by the *Birdsuite *authors, the concepts and approach can be applied to any genotyping array [12] and they are planning on providing support for other high-throughput genotyping platforms, such as the Illumina 1 M.

The *CNGen *algorithm is not limited to the *Fawkes *procedure. As long as the input file format is respected, *CNGen *will conduct the partitioning process. For instance, results from the *PennCNV *software [17] could be used.

The *CNGen *algorithm relies upon the assumption that ancestral copy number expansions are of the same allele type on a given chromosome. In a recent publication by Hastings *et al. *[2], a general overview of the molecular mechanisms of change in gene copy number was presented, owing strong support for the involvement of DNA repair mechanisms which would, in great majority, be concordant with chromosome-specific expansions. There is a range of possibilities however, and copy number expansions occurring during recombination at meiosis, for example, could lead to different allele-type CN expansions. For regions where the assumption of identical allele-type in expansions doesn't hold, the majority will lead to Mendelian inconsistencies following the partitioning algorithm, and will be removed during data quality controls. This will result in a lower number of partitioned genotype for linkage analysis.

Our simulation experiments support the validity of the *CNGen *algorithm and its robustness to *Fawkes *genotype errors and *de novo *mutations.

## Availability and requirements

**Project name**: CNGen

**Project home page**: http://www.statgen.org/ in the download section

**Operating system(s)**: Platform independent

**Programming language**: Python™

**Other requirements**: Standard Python Software 2.5 or 2.6

**License**: none

**Any restrictions to use by non-academics**: none

## Authors' contributions

LPLP worked on the methodology of *CNGen*, implemented *CNGen *and the companion software, performed the validation of the algorithm using simulations and drafted the manuscript. GA participated in the validation of the algorithm and helped to draft the manuscript. GUA participated in the conception of the study and helped to draft the manuscript. MPD conceived of the study, participated in its design and coordination, produced the methodology behind *CNGen *and helped to draft the manuscript. All authors read and approved the final manuscript.

## Supplementary Material

Additional file 1 Archive containing the simulated data for 250 thousand runs (out of 3 million) on a pedigree containing 47 individuals (14 founders). The archive contains the simulated *Fawkes*' calls (file validation.fawkes_calls), the partitioned genotyped compute by *CNGen *and the corresponding log file (file cn_genotype_calls_validation and CNGen.log, respectively) and the pedfile corresponding to the complex pedigree used for simulation (file pedfile.txt). The file (18 Mb) has been uploaded with the present document, and is also available at http://www.statgen.org/main/index.php/Downloads/Downloads. Click here for file

Additional file 2 **thousand validations with errors.tar.bz2**. Archive containing the simulated data for 3 thousand runs with Mendelian errors. The archive contains the same file structure as the first additional file. The data has been splited into three files because of PedCheck's limitations. The file (2.2 Mb) has been uploaded with the present document, and is also available at http://www.statgen.org/main/index.php/Downloads/Downloads. Click here for file
